# Mortality and quality of care in Nordic physician-staffed emergency medical services

**DOI:** 10.1186/s13049-020-00796-9

**Published:** 2020-10-14

**Authors:** Helge Haugland, Anna Olkinuora, Leif Rognås, David Ohlén, Andreas Krüger

**Affiliations:** 1 Department for Research and Development, The Norwegian Air Ambulance Foundation, Postbox 414, Sentrum, 0103 Oslo, Norway; 2 Department of Emergency Medicine and Pre-Hospital Services, St. Olav University Hospital, Trondheim, Norway; 3 Research and Development Unit, FinnHEMS Ltd, Vantaa, Finland; 4 Department of Anaesthesia, Aarhus University Hospital, Aarhus, Denmark; 5 Danish Air Ambulance, Aarhus, Denmark; 6 Airborne Intensive Care Unit, Department of Anaesthesia, Perioperative Management and Intensive Care Medicine, Uppsala University Hospital, Uppsala, Sweden

**Keywords:** Physician-staffed emergency medical service, Helicopter emergency medical service, Mortality, Quality improvement, Quality indicator

## Abstract

**Background:**

Quality indicators (QI) for physician staffed emergency medical services (P-EMS) are necessary to improve service quality. Mortality can be considered the ultimate outcome QI. The process quality of care in P-EMS can be described by 15 response-specific QIs developed for these services. The most critical patients in P-EMS are presumably found among patients who die within 30 days after the P-EMS response. Securing high quality care for these patients should be a prioritized task in P-EMS quality improvement**.** Thus, the first aim of this study was to describe the 30-days survival in Nordic P-EMS as an expression of the outcome quality of care. The second aim was to describe the process quality of care as assessed by the 15 QIs, for patients who die within 30 days after the P-EMS response.

**Methods:**

In this prospective observational study, P-EMSs in Finland, Sweden, Denmark, and Norway registered 30-days survival and scored the 15 QIs for their patients. The QI performance for patients who died within 30 days after the P-EMS response was assessed using established benchmarks for the applied QIs. Further, mean QI performance for the 30-days survivors and the 30-days non-survivors were compared using Chi-Square test for categorical variables and Mann-Whitney U test for continuous variables.

**Results:**

We recorded 2808 responses in the study period. 30-days survival varied significantly between the four participating countries; from 89.0 to 76.1%. When assessing the quality of care for patients who die within 30 days after the P-EMS response, five out of 15 QIs met the established benchmarks. For nine out of 15 QIs, there was significant difference in mean scores between the 30 days survivors and non-survivors.

**Conclusion:**

In this study we have described 30-days survival as an outcome QI for P-EMS, and found significant differences between four Nordic countries. For patients who died within 30 days, the majority of the 15 QIs developed for P-EMS did not meet the benchmarks, indicating room for quality improvement. Finally, we found significant differences in QI performance between 30-days survivors and 30-days non-survivors which also might represent quality improvement opportunities.

## Background

The literature on quality indicators in pre-hospital care is scarce and research initiatives on this topic have been warranted [1, 2]. In a study from 2017 we therefore developed a set of multi-dimensional quality indicators for physician-staffed emergency medical services (P-EMS) through a consensus process. The expert panel agreed on 15 response-specific quality indicators (QIs) for P-EMS; the so called EQUIPE quality indicators [3]. These quality indicators are primarily process indicators; i.e. they describe the process of care provided by P-EMS, rather than the outcome of this care. Process indicators are considered useful for short time frames and when it is difficult to adjust for patient factors [4], and they are therefore particularly relevant for P-EMS. Further, process indicators often provide a more direct measurement of quality of care, whereas structure and outcome indicators often measure this quality more indirectly [5].

The fact that process indicators seem particularly suitable for prehospital services does not make outcome indicators like mortality less important. Mortality within a defined period after hospital admission (commonly 30 days) is considered an appropriate outcome measure for in-hospital care [6]. Some have even argued that outcome indicators are the “ultimate measure of quality in care” [7]. However, for in-hospital care the use of mortality as a quality measure has been questioned because the number of patients that die, or at risk of dying, are actually fairly low, thus making mortality less suitable as a quality measure [8]. Hospital mortality has also been used when assessing the effects of pre-hospital care. A paradox is that outstanding pre-hospital care in fact may increase hospital mortality because patients survive until hospital admission rather than die on scene or en route [9, 10].

A widely cited definition of quality that also might be applicable for P-EMS systems is “the degree to which health services for individuals and populations increase the likelihood of desired health outcomes and are consistent with current professional knowledge” [11]. This definition supports the idea that outcome alone is not sufficient to describe the total quality of care. However, it seems reasonable that good processes ultimately lead to better outcome. This principle is used in other high-risk businesses as well; in aviation, petroleum industry and nuclear power plants for instance, the process quality is measured – assuming that good process quality will prevent a major incident [12]. In medical research, however, the use of hard end points, especially mortality, has been the gold standard. Yet, a study on the relationship between quality and mortality for acute hospitals in England concluded that high mortality was not an adequate marker of overall poor quality [13]. Nevertheless, mortality is an undeniable quality indicator in pre-hospital care and knowing the survival rate of P-EMS therefore seems highly relevant.

In this study, we aimed to explore the 30-days survival in Nordic P-EMS as an expression of the outcome quality of care. Further, we aimed to describe the process quality of care as assessed by the EQUIPE quality indicators for these patients who die within 30 days after the P-EMS response.

## Methods

### Study design and setting

In this prospective observational study, 16 physician-staffed helicopter emergency services in Finland, Sweden, Denmark, and Norway registered data for the EQUIPE quality indicators. Additionally, 30-days mortality data was collected for all included patients. There has previously been documented significant system similarities in the P-EMS of the four participating countries making them a suitable arena for multi-centre studies [14]. All services respond to pre-hospital patients (primary responses), and the Swedish, Danish, and Norwegian services also do transfers between hospitals regularly. Finnish P-EMS do inter-hospital transfers only by exception. Moreover, the Norwegian services also do search and rescue responses (SAR-responses). In addition, one Swedish (Karlstad) and all Finnish and Norwegian bases dispose a rapid response car for responses close to the base and for responses in poor weather conditions that prevent flight operations.

As a framework for this paper, we have used the Strengthening the Reporting of Observational studies in Epidemiology (STROBE) guidelines [15].

### Inclusion criteria and data variables

All P-EMS requests were included in the study. Thus, we could include both completed and cancelled responses as well as stand-downs (responses cancelled by dispatch or crews on-scene) and rejected responses. Examples of reasons for rejecting a response might be weather conditions or the lack of medical need as judged by the P-EMS physician. The latter is possible in Sweden, Finland, and Norway where the acceptance or rejection of a response is at the P-EMS physicians’ discretion. Inquiries with the provision of telemedical advice only were excluded.

For the analysis of time variables, inter-hospital transfers and SAR-responses were omitted, as the nature of these responses is not comparable to primary responses pertaining to time consumption.

### Data sources/measurement

The Swedish, Danish and Norwegian services registered the data by using a web-based questionnaire (Formsite; Vroman systems, Inc., Chicago, Illinois). The Finnish HEMS collected the necessary data by including the quality indicators as part of their existing documentation database (FinnHEMS database, FHDB). FHDB is a national database, including both response and patient data, where all HEMS units register their responses. Some QIs could be gathered from the existing data, other QIs were either implemented as permanent variables or on a separate study sheet. Filling in all QIs was mandatory.

In all countries the data were collected after completed response by the P-EMS physician. Four national investigators performed data quality assurance and collected 30-days survival in their respective countries. Data for the QIs was collected for 3 months, followed by collection of 30-days survival data. The total data collection period was July 2016 – April 2017.

### Statistical methods

Results are presented using descriptive statistics. The QI proportions were recorded for QIs that are categorical variables; time was recorded in minutes for QIs that were continuous time variables. All quality indicators are reported by their mean and the corresponding 95% confidence interval. For the purpose of describing the quality of care for patients who died within 30 days, a quality scale for the EQUIPE quality indicators was used. This quality scale presents QI performances as average (within the interquartile range (IQR); yellow zone), above average (above IQR; green zone) or below average (below IQR; red zone) [16] based on the value of all QIs. Moreover, the quality scale defines a benchmark for every QI at the transition between the yellow and green zone. To explore a possible difference in quality indicator score between the groups “Alive after 30 days” and “Dead after 30 days”, the mean values for each separate QI are compared using Chi-Square test for the categorical variables and Mann-Whitney U test for the continuous variables due to non-normality. Defined significance level is *p* < 0.05.

### Missing data

Responses with missing data pertaining to 30-days survival are omitted from the analysis.

## Results

### Participants and descriptive data

**Fig. 1:**
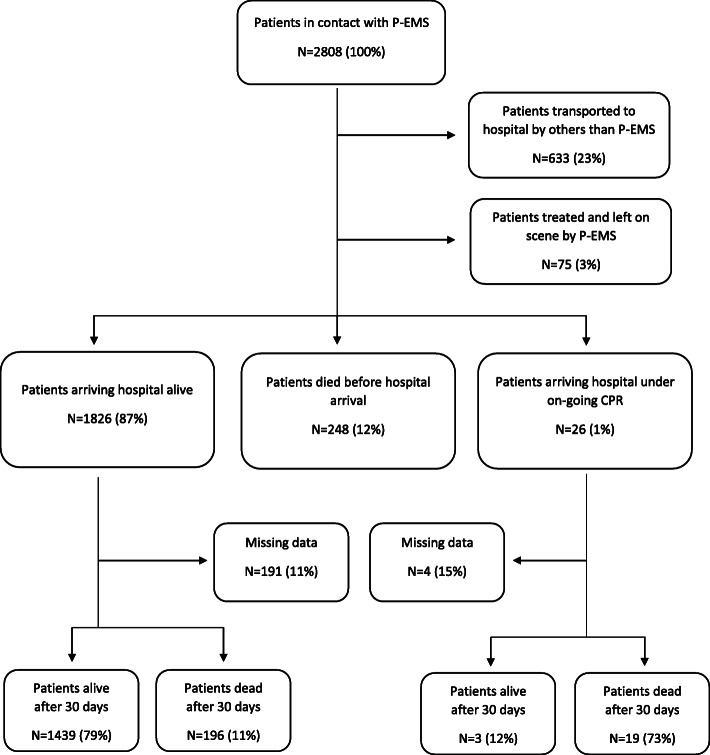
Flowchart of the study. “Treat and leave” = patients left on scene and not going to hospital

The dataset consisted of 2808 patients in contact with P-EMS. The patient flow and survival from these requests is depicted in Fig. 1. Finland recruited 37% of the patients, Norway 28%, Denmark 24% and Sweden 11%.

### Outcome data and main results

Of the 2808 patients cared for by P-EMS a total of 633 (22.5%) patients were eventually transported to hospital by other services than P-EMS. 525 (82.9%) of these patients were still alive 30 days after the P-EMS response, 45 (7.1%) were dead and data were missing for 63 patients (10.0%).

**Fig. 2:**
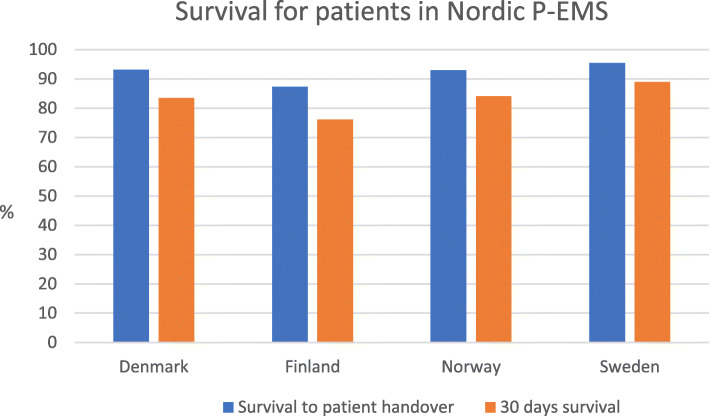
Survival for patients in Nordic P-EMS

In Fig. 2 “Survival to patient handover” and “30-days survival” is depicted for all four participating countries. Survival to patient handover is defined as survival until the patient is handed over in the hospital or as survival until handover to EMS when transported by others than P-EMS. Survival to patient handover was 93.2% (Denmark), 87.3% (Finland), 93.0% (Norway) and 95.5% (Sweden). The proportion of patients surviving until 30 days after the actual P-EMS response was 83.5% (Denmark), 76.1% (Finland), 84.1% (Norway) and 89.0% (Sweden), respectively. The difference between Finland and Sweden had a *p*-value < 0.00.

**Fig. 3:**
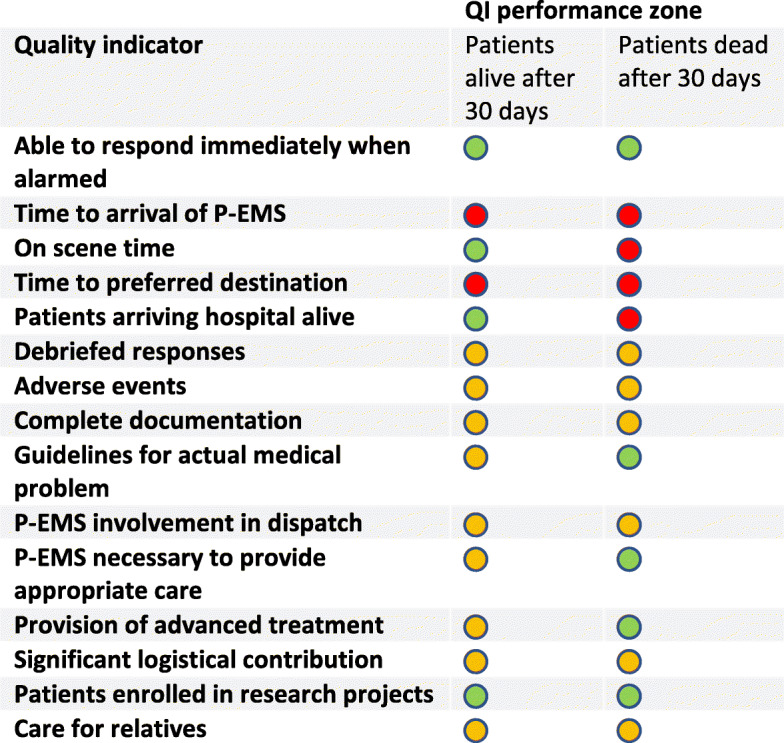
Quality indicator performance using the EQUIPE quality scale and benchmarks. Green zone, high performance; yellow zone, medium performance; red zone, low performance. The benchmark is set at the transition between green and yellow zones. Thus, performances in the green zone meet the benchmark. Time variables are presented as medians as they are not normally distributed. The remaining QIs are presented as means of proportions

In Fig. 3, the QI performances are depicted for patients alive or dead 30 days after the P-EMS response, respectively. For the patients who died within 30 days after the P-EMS response, four QIs are within the red zone of performance, indicating a performance below medium quality. Six QIs are within the yellow zone of performance according to the EQUIPE quality scale, indicating a performance of medium quality. Finally, five QIs are within the green zone of performance, indicating a performance above medium quality. Thus, these are the only five QIs which meet the benchmark as defined by the EQUIPE quality scale.

For all 15 QIs, comparisons of QI scores between the 30-days survivors and 30-days non-survivors are presented in Table 1. For nine out of 15 QIs, there was significant difference in mean QI scores between the survivors and non-survivors.

**Table 1:** Quality indicator performance for patients surviving and not surviving 30 days after the P-EMS response

Quality indicator	Unit	Alive after 30 days (***n*** = 2293) mean (95% CI)	Dead after 30 days (***n*** = 521) mean (95% CI)	p-value
Ability to respond immediately when alarmed	%	95 (94–96)	95 (95%CI: 93–97)	0.226
Time to arrival of P-EMS	minutes	33 (31–34)	30 (28–32)	0.106
On scene time	minutes	19 (18–20)	29 (27–30)	0.000
Time to preferred destination	minutes	83 (76–89)	79 (68–89)	0.542
Survival to hospital	%	100 (100–100)	54 (50–58)	0.000
Debriefed responses	%	71 (69–73)	73 (70–77)	0.293
Adverse events	%	2 (1–2)	3 (1–4)	0.054
Complete documentation	%	64 (62–66)	74 (70–78)	0.000
Guidelines for actual medical problem	%	58 (55–60)	78 (74–82)	0.000
P-EMS involvement in dispatch	%	44 (42–46)	37 (33–41)	0.000
P-EMS necessary to provide appropriate care	%	35 (33–37)	55 (51–60)	0.000
Provision of advanced treatment	%	42 (40–44)	76 (73–80)	0.000
Significant logistical contribution	%	44 (42–46)	27 (23–31)	0.000
Patient enrolment in research projects	%	7 (6–9)	11 (8–14)	0.013
Care for relatives	%	93 (92–95)	95 (92–97)	0.413

## Discussion

In this prospective observational study, 30-days survival in P-EMS patients varied significantly between the four participating countries; from 89 to 76%. For the patients who died within 30 days, the quality of care, as assessed by the EQUIPE quality scale, met the benchmark for five out of 15 quality indicators. For nine out of 15 QIs we found significant differences in QI score between 30-days survivors and 30-days non-survivors. Based on the results of this study, we consider there to be room for quality improvement for patients cared for by P-EMS.

Mortality measures are easy to define and have traditionally been important in reducing preventable deaths in health care. A major strength of mortality as quality indicator is the fact that it is a hard outcome - as well as it’s undisputable importance for the patients and their relatives. Nevertheless, mortality is not necessarily an optimal QI. The rate of preventable deaths have decreased due to improved care, and death simply does not occur often enough in some patient groups to secure the necessary frequency of an event for it to be a meaningful QI [17, 18]. In a Danish study, 30-days mortality for pre-hospital patients varied between 2.3% (Trauma) and 49.3% (Unconsciousness/Cardiac arrest) [19]. Thus, mortality should not stand alone as QI, but be part of a comprehensive quality measurement approach, as one of several quality indicators. As such, the quality of care for all pre-hospital patients should also be measured by process measures, because outcome and process quality are two different concepts. A patient might receive state-of-the-art care, but still die due to the severity of the disease or trauma. In such a case, using mortality alone will not reflect the high quality in the process of care. Opposite, a patient might receive poor care but still survive. In a case like that, using mortality as the only QI will not reflect the low quality in the process of care. Measurements of mortality and process quality are complementary, and both are central to identify the total quality achieved in a system. We argue that our findings underline these statements, as there was no difference in QI performance between survivors and non-survivors for six out of 15 QIs. Moreover, for the nine QIs where a significant difference was found, the results seem to be explained first and foremost by presumably more complex and critical conditions for the non-survivors, requiring more advanced treatment on scene. However, the differences between the two patient groups might represent possible areas for quality improvement initiatives.

We found that survival to patient handover varied significantly across countries. Survival until 30 days after the P-EMS response was also different. For both variables, Sweden has the lowest mortality and Finland has the highest mortality in this study. This may be a reflection of different use of the P-EMS units. In Finland, the proportion of inter-hospital transports is lower than in the other countries; 3.1% (F) vs 20.4%(DK), 34.9%(S) and 41.6%(N). These differences in use may be contributing to the different mortality numbers because patients transported between hospitals normally are in a more stable phase than the patients cared for in primary responses. Another possible explanation may be that Finnish P-EMSs are dispatched to more critical patients than in the other Nordic countries.

For patients who died within 30 days, the QIs “Time to arrival of P-EMS” and “Time to preferred destination”, both had a QI performance below average according to the EQUIPE quality scale. A core task for P-EMS is to bring the hospital competence to the pre-hospital patient to ensure that critical care can start at an earlier stage. This has been documented to improve outcome or at least physiological variables in selected patient groups [9, 20, 21]. Furthermore, bringing the patient swiftly to the preferred destination to provide definitive care is critical for conditions like acute myocardial infarction, ischemic stroke and major trauma [22]. Both aforementioned QIs are time variables primarily dependent of the infrastructure of the P-EMS system, including P-EMS base distribution. Thus, meeting the benchmark for these QIs probably would require changes of infrastructure, but may also stimulate the innovation of solutions bringing definitive care to the patient’s location.

When exploring the difference in QI performance between the 30-days survivors and 30-days non survivors, we found that the on-scene time was significantly higher for the latter patient group. A possible explanation for this might be that these patients presumably are in a more critical condition when P-EMS arrives, and that this necessitates time-consuming interventions. This finding is supported by the significantly higher proportion of advanced interventions in the group of non-survivors. Some might argue that this difference implies that long on-scene time is harmful for severely injured or ill patients. However, there is no adequate basis for drawing conclusions regarding causality in this study, only conclusions regarding correlation. Complete documentation was found more frequently in the group of non-survivors. This might be because the P-EMS physician feels a greater need for both obtaining and documenting clinical parameters when the patient is severely ill. Nevertheless, the defined key parameters should be relevant documentation for all patients [23]. Also the presence of guidelines was significantly higher in the group of non-survivors, probably due to more established guidelines for the most critical conditions - as a timely and efficient approach is of paramount importance in these situations. Involvement of the P-EMS physician to decide if dispatch is appropriate occurred more often in the group of survivors. This may indicate that the alarm calls to EMCC for the most severely ill or injured patients leave less doubt regarding the dispatch of P-EMS, while patients of lower severity are discussed more frequently with the P-EMS physician prior to dispatch. The proportion of responses in need of P-EMS to secure appropriate care was significantly higher in the group of non-survivors. This could be due to the higher need for advanced interventions and critical decision making in this group.

The quality measurement model used in this study allows P-EMS to identify and monitor variations in their services. Reducing variation is considered imperative in quality improvement [24]. In this study, the difference in necessary documentation for 30-days survivors and 30-days non-survivors is an example of variation. Our model of combining mortality and QIs are intended to be used as a means to identify areas for improvement; expressed as unwanted variation. When process quality variation is identified, different approaches exist to obtain more standardisation. In the example above, possible approaches to improve the documentation quality could be to change the patient documentation systems, improve the registration practice, introduce more automatized documentation etc. Moreover, the quality measurement model enables comparisons with established benchmarks. Thus, suboptimal QI performances can be identified and necessary quality improvement initiatives can be established. In our study for instance, identifying that benchmarks are not met for the QI “Time to arrival of P-EMS” for patients who died within 30 days, may lead to directed quality improvement projects. More P-EMS bases, changing the existing P-EMS base locations and even better coordination of neighbouring P-EMS resources may be different ways of reducing time to arrival of P-EMS.

### Limitations

Mortality is influenced by the patient’s actual diagnosis and comorbidity. Nonetheless, we have included all patients when exploring the mortality in Nordic P-EMS. This was done to secure a normal clinical setting in P-EMS. Moreover, the EQUIPE QIs used to describe the quality of care of patients in this study are developed for everyday quality measurement in international P-EMS regardless of patient characteristics. Hence, this seems to be the adequate setting for our study. However, it might be that subgroup analysis on specific patient groups, for instance high mortality diagnosis, would reveal different mortality rates and even different QI performances for these subgroups.

Regarding missing data, “Survival to handover”-data were missing for only 6 out of 2814 patients. However, 30-days survival data were missing for 9.7% of the patients. These are either patients with foreign personal identification number or patients with unknown identity. Both patient groups are taken care of regularly by P-EMS. The problem of losing patients to follow-up because of unknown identity in the pre-hospital phase has also been reported by Christensen et al., who reported a loss to follow-up of 17.8% [25]. In all four countries we experienced the same difficulties pertaining to these patient groups when collecting 30 days survival data. The data collection period was partly in the summer months, when the number of foreign tourists in the Nordic countries is high. Thus, the proportion of missing data might at least partly be explained by a relatively high number of foreign citizens treated by P-EMS. We have no reason to believe that the mortality of the mentioned patient groups differs significantly from the rest of the patient cohort. Thus, we assess the 30 days survival figures as representative for the patients in the study group, although the missing data for these figures ideally should be lower to secure the most valid results.

It is also vital to emphasize that this being an observational study we are in no position to suggest a causative correlation between QIs and outcome; we are merely highlighting that there seems to be an association between some of the QIs and mortality.

## Conclusion

In this study we have explored the mortality in Nordic P-EMS. 30-days survival varied significantly between the four participating countries; from 89.0 to 76.1%. Furthermore, we have assessed the quality of care for patients who die within 30 days after the P-EMS response. Only five out of 15 QIs met the benchmark for this patient group, indicating a potential for quality improvement initiatives. When comparing QI performances between 30-days survivors and 30-days non-survivors, we found significant differences for nine out of 15 QIs. These differences could, at least partly, be due to presumably more complex and critical conditions for the non-survivors, but the differences between the two patient groups might represent possible areas for quality improvement initiatives.

## Data Availability

The datasets analysed during the current study are available from the corresponding author on reasonable request.

## Abbreviations

EMS: Emergency medical service
P-EMS: Physician-staffed emergency medical service
HEMS: Helicopter emergency medical service
SAR: Search and rescue
QI: Quality indicator
EQUIPE: Establishing quality indicators in physician-staffed emergency medical services
